# Targeted insertion of large genetic payloads using cas directed LINE-1 reverse transcriptase

**DOI:** 10.1038/s41598-021-03130-0

**Published:** 2021-12-08

**Authors:** Femila Manoj, Laura W. Tai, Katelyn Sun Mi Wang, Thomas E. Kuhlman

**Affiliations:** 1 Microbiology Program, University of California Riverside, Riverside, CA 92521 USA; 2 Department of Biology, University of California Riverside, Riverside, CA 92521 USA; 3 Department of Physics and Astronomy, University of California Riverside, Riverside, CA 92521 USA

**Keywords:** Biological techniques, Genetic engineering

## Abstract

A difficult genome editing goal is the site-specific insertion of large genetic constructs. Here we describe the GENEWRITE system, where site-specific targetable activity of Cas endonucleases is coupled with the reverse transcriptase activity of the ORF2p protein of the human retrotransposon LINE-1. This is accomplished by providing two RNAs: a guide RNA targeting Cas endonuclease activity and an appropriately designed payload RNA encoding the desired insertion. Using *E. coli* as a simple platform for development and deployment, we show that with proper payload design and co-expression of helper proteins, GENEWRITE can enable insertion of large genetic payloads to precise locations, although with off-target effects, using the described approach. Based upon these results, we describe a potential strategy for implementation of GENEWRITE in more complex systems.

## Introduction

Discovered as a bacterial immune system against foreign genetic elements such as phages, CRISPR (Clustered Regularly Interspaced Short Palindromic Repeats) Associated Proteins (Cas) are endonucleases that target and cleave DNA sequences based upon their homology with a “guide RNA”^1,2^. Consequently, by providing an engineered “single-guide” RNA (sgRNA), Cas enzymes can be targeted to cleave any desired sequence. This flexibility in gene editing by CRISPR-Cas endonucleases has revolutionized genome editing^3–6^ in a wide variety of organisms^7–23^ and in its application to clinical therapeutics^25^.

Despite their flexibility and ease of use, the repertoire of genome editing modalities that CRISPR/Cas systems allow remains limited. Knockout or point mutants can be generated relatively easily by targeting Cas cleavage to coding or control regions of the genome. The cell must repair such cuts to survive, and errors introduced by the nonhomologous end joining (NHEJ) repair machinery can lead to inactivation of control regions or introduction of missense or point mutations to coding sequences^26–29^. An additional editing modality is to introduce novel sequences to the genome through Homology Directed Repair (HDR), where a DNA fragment with ends homologous to the sequences flanking the cut site and containing the desired sequence to be inserted is introduced to the cell along with the Cas-sgRNA ribonucleoprotein (RNP) complexes. After cleavage, the fragment is then used to repair the cut by the cell’s homologous recombination repair machinery, resulting in its integration. However, HDR remains inefficient and difficult to accomplish, particularly for gene-sized or larger [≥ ~ 1 kilobase pair (kbp)] fragments^30–33^. A primary reason for this difficulty is that for HDR to be successful, non-homologous end joining (NHEJ) DNA repair, the primary repair mechanism for DNA repair in advanced eukaryotic cells^34–37^, must be suppressed^38–40^.

**Figure 1:**
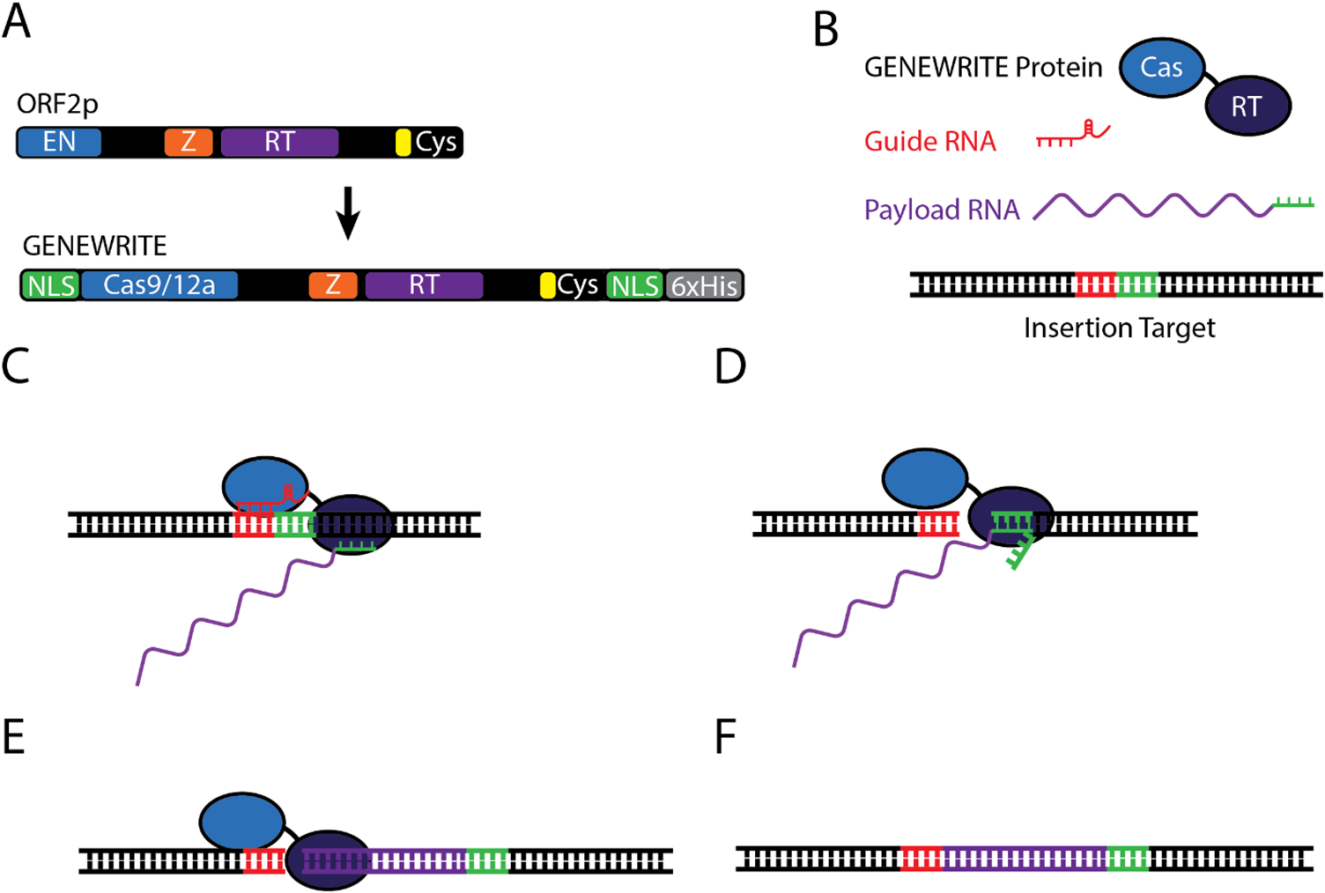
GENEWRITE components and strategy. (**A**) ORF2p and GENEWRITE domain structure. Wildtype ORF2p consists of endonuclease (EN, blue), Z (Z, orange), reverse transcriptase (RT), and cysteine-rich RNA binding domains (Cys, yellow). The GENEWRITE protein replaces the EN domain with a Cas protein (Cas9 or Cas12a/Cpf1, blue) and includes an N-terminal EGL13 nuclear localization signal (NLS, green), C-terminal c-Myc NLS (NLS, green), and 6xHis tag for in vitro purification (His, gray). (**B**) GENEWRITE components. The system consists of the GENEWRITE protein and a DNA target for insertion. A guide sgRNA complementary to the desired cut site (red) and a payload RNA encoding the desired insertion with a 3’ end designed to hybridize to the insertion target (green). Optionally, as described in the text, NHEJ proteins, ORF1p protein, and 5’ homology on the payload RNA to the target site can be included to increase insertion efficiency. (**C**) The sgRNA directs Cas cleavage to the integration site. (**D**) After Cas-induced cleavage, the 3’ end of the payload RNA hybridizes with the cut site priming TPRT (**E**). After mRNA removal and second strand synthesis by host enzymes, the cut site is resolved (**F**).

Here we introduce a method for the active insertion of lengthy genetic sequences into host DNA we call GENEWRITE: Genome Engineering With RNA-Integrating Targetable Endonucleases. This is accomplished by coupling the targetable endonuclease activity of Cas enzymes to the reverse transcriptase activity of the human retrotransposon LINE-1 through translationally fusing Cas and LINE-1 reverse transcriptase proteins (Fig. 1A). A number of recent reports have described approaches coupling the targetability of Cas enzymes with the activity of other transposons or reverse transcriptases. These include Tn7-like transposons whose genomic insertion is accomplished through an associated CRISPR-effector, from the cyanobacterium *Scytonema hofmanni*^41^ and *Vibrio cholerae*^42^. Insertion of these 2–3 kbp bacterial transposons is programmable to specific genomic locations in *E. coli* through a guide RNA similar to other Cas enzymes. Another approach, prime editing^43^, fuses a catalytically impaired Cas9 fused to an engineered Moloney Murine Leukemia Virus (M-MLV) reverse transcriptase^44–47^, using a “prime editing guide RNA” (pegRNA) to target short insertions, deletions, and all types of point mutations into human cells. GENEWRITE offers functionality that is distinct from each of these examples. While prime editing similarly uses a reverse transcriptase to insert RNA-encoded sequences into the genome, insertions performed with prime editing are typically limited to short 10–40 bp epitopes. Conversely, we illustrate the site-specific reverse transcription and insertion of ~ 1.5 kbp payload RNAs, larger than that offered by prime editing.

Previous studies have shown that reverse transcription by the LINE-1 protein ORF2p can be directed to pre-existing nicks and cuts in targeted DNA sequences in vitro^48^, and we have previously shown that LINE-1 is functional in *E. coli*, particularly when complemented by expression of enzymes for NHEJ repair^49^. Here we use *E. coli* expressing *B. subtilis* NHEJ enzymes as a simple platform to optimize design and delivery of GENEWRITE for future application to more complex systems. The strategy used here for integration using GENEWRITE is shown in Fig. 1B–F. *E. coli* cells expressing GENEWRITE are transformed with two RNAs: a guide sgRNA to target Cas cleavage to the desired integration site, and a payload RNA carrying the coding sequence of the desired integration. The 3’ end of the payload RNA is designed to be homologous to the bottom DNA strand downstream from the cut such that RNA–DNA hybridization occurs to prime reverse transcription. Host enzymes complete second strand synthesis and payload RNA removal, and the insert is sealed into the site^49^. We illustrate that GENEWRITE can be used to effectively target insertion of large, gene-sized payloads to specific locations, although not without off-target effects.

## Results

### GENEWRITE rationale and design

The human retrotransposon LINE-1 (Long Interspersed Nuclear Element, or L1) encodes the two proteins ORF1p and ORF2p, and both proteins are required for efficient retrotransposition in humans. A primary function of ORF1p appears to be chaperone activity^50^, while ORF2p includes endonuclease (EN) and reverse transcriptase (RT) domains. To retrotranspose, ORF2p EN nicks TA-rich target DNA, and the 3’ end of the LINE-1 mRNA hybridizes with DNA adjacent to the nick to initiate reverse transcription through a process called target primed reverse transcription (TPRT)^51^. In most active L1 elements, this hybridization is facilitated through the presence of a ~ 100 bp long poly(A) tract, which is also thought to be the primary binding target of ORF2p to its encoding mRNA^52^.

LINE-1 and its accessory proteins naturally exist in human cells, making it an appealing target for optimization as a genome editing tool. To attempt to further enhance specifically targeted reverse transcribed insertions by ORF2p in vivo, we removed the promiscuous ORF2p EN domain by deleting amino acids 1–347. The remaining fragment, from amino acids 348–1275, which includes the Z, RT, and cysteine-rich RNA-binding domains, we dub ORF2pZRT. Finally, the GENEWRITE protein consists of a translational fusion of ORF2pZRT to targetable Cas endonucleases (Cas9 or Cas12a/Cpf1) with a flexible linker. In addition, the GENEWRITE protein includes N and C-terminal nuclear localization signals (NLS) and a C-terminal 6xHis tag to enable purification (Fig. 1A). A previous similar attempt at replacing ORF2p EN with Cas9 and using *Alu*-like payload RNA to target ORF2p RT to specific loci in human cells proved unsuccessful^53^. As described below, we have made several refinements to the GENEWRITE system relative to this attempt, including the use of *Escherichia coli* as a simpler in vivo platform in which to test and optimize. We additionally show that the 10 base pair homology between target and payload used in this previous study is likely inadequate for priming of TPRT.

### High expression of GENEWRITE protein is Lethal to E. coli

We designed and synthesized the GENEWRITE protein under control of a T7 promoter, which was cloned into the plasmid pUC57-*kan*. We transformed this plasmid into *E. coli* strain BL21-AI, along with either empty plasmid pZA31, or pZA31 carrying *ykoU* and *ykoV B. subtilis* NHEJ enzymes expressed from P_LtetO1_^49,54^. In strain BL21-AI, GENEWRITE expression is inducible by the addition of L-arabinose. Curiously, while expression of Cas9/12a, ORF2pZRT, or both Cas9/12a and ORF2pZRT in individual *E. coli* cells does not affect growth, strong expression of the GENEWRITE Cas-ORF2pZRT fusion protein induced through the addition of arabinose is lethal to *E. coli*. This lethality is partially relieved by simultaneous expression of *B. subtilis* NHEJ enzymes. This suggests lethality may be a consequence of genomic breaks generated by GENEWRITE, perhaps driven by high affinity of ORF2p to arbitrary RNAs in vivo^55^. Consequently, the results described below rely upon low, leakage levels of expression of GENEWRITE without induction.

### GENEWRITE is effective at insertions into high-copy number targets in E. coli

We expected the strategy outlined in Fig. 1B–F to be difficult to successfully execute for a number of reasons, including the expected difficulty of co-transforming individual cells with appropriate amounts of both sgRNA and payload RNA, as well as previously documented preference of ORF2p to act primarily upon its *cis*-encoding RNA^56^. Hence, as an initial integration target, we chose the high copy number plasmid pUC57-*kan* [~ 500–1000 /cell] from which the GENEWRITE protein itself is expressed to maximize chances of success. For experiments described here, the ~ 1200 bp payload RNA consisted of an *aadA* spectinomycin resistance gene driven by a strong, constitutive *lacI*Q1 promoter^57^ and Shine-Dalgarno ribosomal binding site (RBS). Consequently, after the GENEWRITE protocol, cells were spread on plates containing spectinomycin to select for potentially successful integrants.

Based upon our current understanding of TPRT, design of the payload RNA 3’ hybridization region is critical. To determine the optimal length of the hybridization region, we generated an array of six identical payload RNAs with hybridization length variable from 0 to 50 bp in 10 bp increments. Based on prior reports of the essentiality of a 3’ poly(A) tract for ORF2p binding and reverse transcription^52^, we generated a second array of payload RNAs, identical to the first, but also including the 30 bp poly(A) tract found in the SINE element AluYA5^58^.

**Figure 2:**
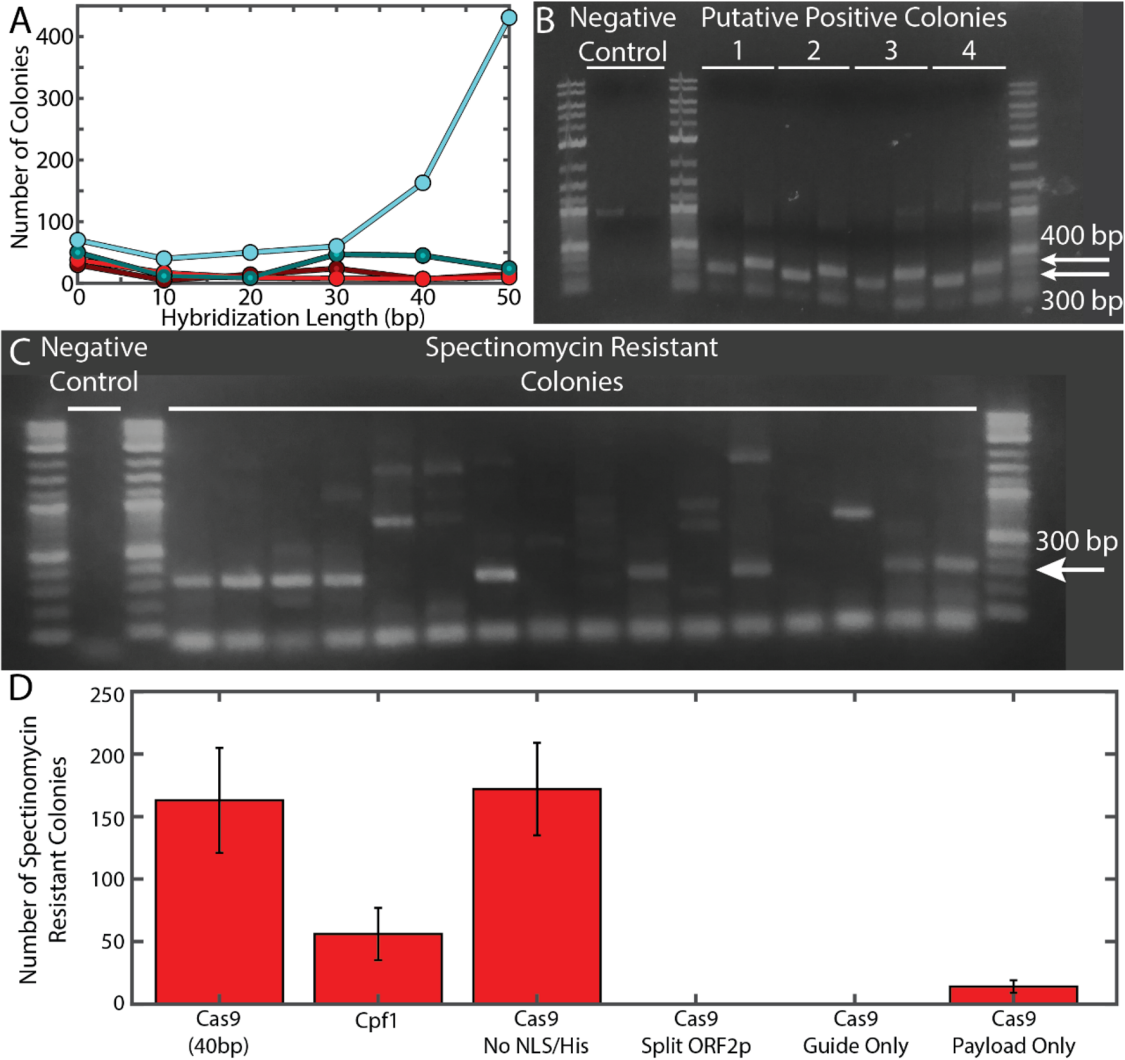
GENEWRITE site-specific insertion in a high-copy number plasmid. sgRNA and payload RNAs were designed to integrate an *aadA* spectinomycin resistance gene into the plasmid pUC57-*kan*. (**A**) Number of spectinomycin resistant colonies as a function of payload RNA 3’hybridization length. Dark red: -NHEJ + poly(A); Dark cyan: -NHEJ -poly(A); Bright red: + NHEJ + poly(A); Bright cyan: + NHEJ -poly(A). Data points are the average of three replicates, error bars are SD. (**B**) PCR verification of integration. Lanes 1, 4, 13: NEB 1 kb Plus Ladder. 300 and 400 bp bands are indicated. Lanes 5–12: amplicons resulting from four spectinomycin resistant colonies. For each pair of lanes, the leftmost lane is PCR across the 5’ junction (300 bp amplicon expected), rightmost is PCR across 3’ junction (400 bp amplicon expected). (**C**) Representative screening of 16 randomly selected colonies by PCR across the 5’ integration junction. (**D**) Controls and effect of various GENEWRITE components, relative to that of intact GENEWRITE-Cas9 with 40 bp homology payload RNA (first column). 2nd column: replacement of Cas9 with Cas12a/Cpf1; 3rd column: effect of removal of NLS and His tags; 4th column: simultaneous expression of unfused Cas9 and ORF2pZRT; 5th column: GENEWRITE-Cas9 transformed with only guide RNA but no payload; 6th column: GENEWRITE-Cas9 transformed with only payload RNA but no guide.

We transformed the pUC57-targeting sgRNA along with each payload RNA into *E. coli* weakly expressing GENEWRITE-Cas9, either with or without simultaneous expression of *B. subtilis* NHEJ enzymes. The results are shown in Fig. 2. For those payload RNAs containing a poly(A) tract, we observed very few spectinomycin resistant colonies, for both with or without simultaneous co-expression of NHEJ. Conversely, without the poly(A) tract, we obtained hundreds of spectinomycin resistant colonies when complemented with co-expression of NHEJ (Fig. 2A). Site-specific integration was verified by PCR using primers that amplified across the 5’ and 3’ integration junctions (Fig. 2B); 63 out of 96 colonies screened yielded a positive signal for a success rate of ~ 72% (Fig. 2C). Sequencing of eight purified plasmids revealed some small deletions at the 5’ end of the insertion (Supplementary Fig. S1). From these experiments, we conclude that optimal design of the payload RNA includes 40–50 bp of 3’ homology to the intended target facilitated by NHEJ DNA repair, and with no poly(A) tract. This difference in essentiality of the poly(A) tract to TPRT between *E. coli* and humans may be the result of mRNA 3’ poly(A) tails stabilizing RNAs in eukaryotes, while poly(A) tails designate mRNAs for degradation in bacteria^59–62^.

We performed a series of controls and further investigations using the payload designed to target pUC57-*kan* with 40 bp 3’ hybridization region (Fig. 2D): (1) as expected, the sgRNA is required for efficient targeting and integration; (2) NLS sequences at the N- and C-termini do not significantly interfere with function; (3) the Cas12a/Cpf1 GENEWRITE variant is functional, although with lower efficiency than the Cas9 variant, consistent with previous findings that blunt-end cuts fragments serve as better TPRT substrates than those with 3’ or 5’ overhangs^48^; and (4) simultaneous co-expression of unfused Cas9 and ORF2pZRT, rather than the translationally-fused GENEWRITE protein, is not functional. However, LINE-1 reverse transcriptase has been shown to function even when encoded and expressed separately from the endonuclease through association via the naturally occurring cryptic Z domain, raising the possibility of potentially using naturally expressed LINE-1 in the human genome as an editing tool.

### GENEWRITE can insert payloads into low and single copy targets

**Figure 3:**
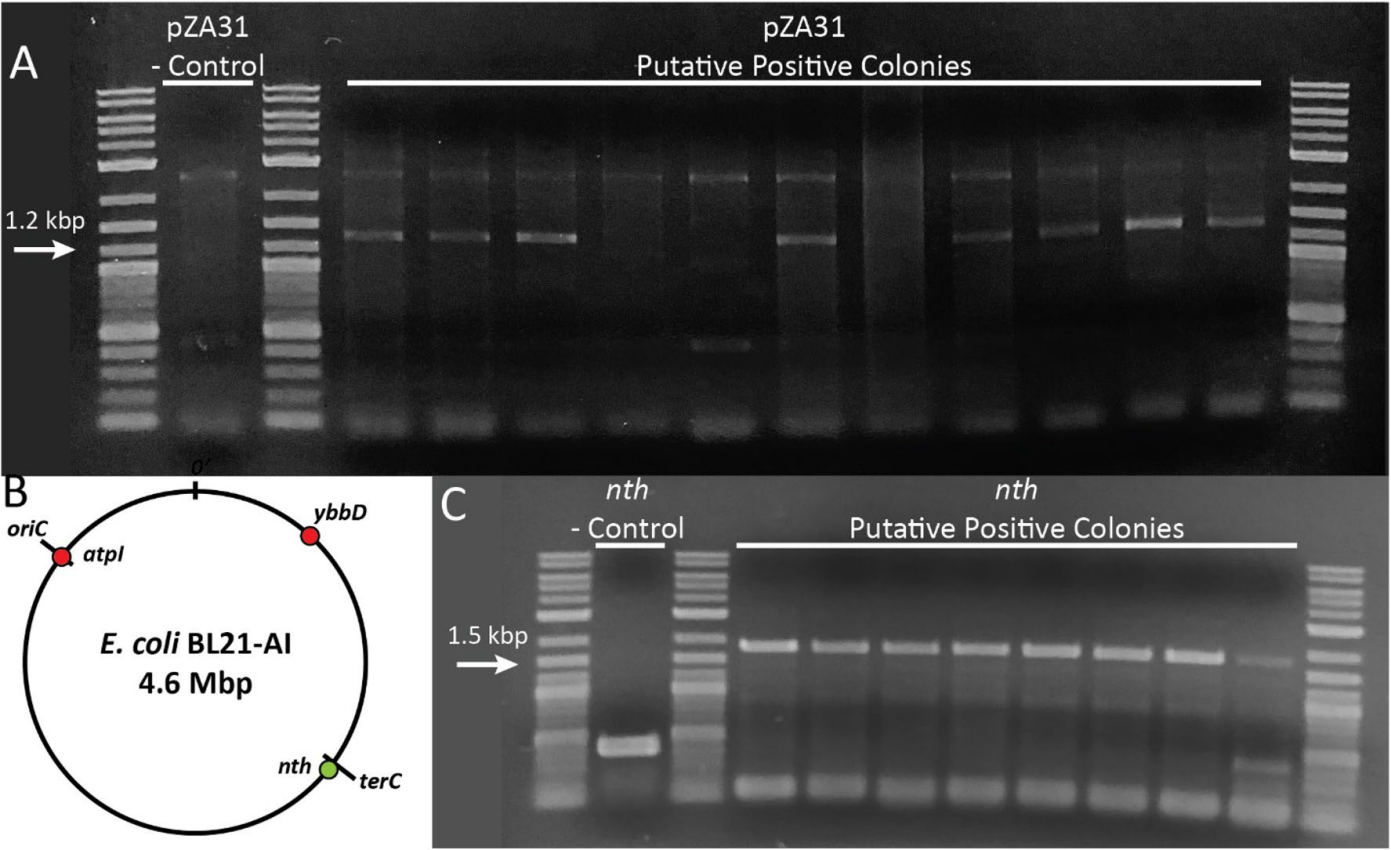
GENEWRITE site-specific insertion in a low-copy number targets. (**A**) Insertion in low copy number (15–30 copies /cell) plasmid pZA31. Chosen primers bind at start of payload promoter and within the adjacent pZA31 sequence after 3’ end of payload. Colony PCR was performed with 10% DMSO to eliminate extraneous non-specific amplification. Expected amplicon is ~ 1300 bp. (**B**) Attempted chromosomal insertion sites. Coordinates of sites are $x_{chromosome}$ = 0.0098 (*atpI*), 0.5476 (*ybbD*), and -0.943 (*nth*), where *x* = 0 corresponds to *oriC* and *x* =  ± 1 corresponds to *terC*. (**C**) PCR amplification across *nth* integration location. Primers bind to chromosomal regions adjacent to targeted integration site. Amplicon expected from successful integration is ~ 1600 bp. We conservatively identify the last colony as negative for integration.

For the next target, we attempted insertion into the much lower copy number pZA31 plasmid hosting the NHEJ genes [~ 20–30 copies/cell^54^]. Using 40 bp of 3’ homology to target as described above, we obtained no colonies when transforming payload and sgRNAs into cells expressing GENEWRITE but deficient in NHEJ. However, we obtained ~ 50 colonies on average when transforming into cells expressing both GENEWRITE and NHEJ proteins. PCR screening of putative positive colonies generated a positive signal in 10 out of 50 colonies (Fig. 3A), yielding a success rate of 20%.

We finally attempted to use GENEWRITE to site-specifically insert a payload into single copy chromosomal loci. We attempted insertions at three loci we have previously shown to accept insertions at high efficiency using recombineering-like methods: the *nth* locus near the terminus of replication; the *atpI* locus near the origin of replication; and the *ybbD* locus midway on the right replichore [Fig. 3B^63,64^]. In these cases, repeated attempts at the GENEWRITE protocol as described above were unsuccessful. Prior reports^65^ and our own studies of retrotransposition of native LINE-1 in *E. coli* (Supplementary Fig. S2) suggest that homology between the 5’ end of the payload and insertion location may also aid in targeting. Consequently, we attempted two strategies: (1) inclusion of 20 bp of 5’ homology between the payload and the targeted insertion site; and (2) simultaneous co-expression of ORF1p. Each of these strategies alone was unsuccessful. Only when targeting *nth* by including 20 bp of 5’ and 40 bp of 3’ payload homology to the target, along with simultaneous co-expression of ORF1p, did we obtain significant numbers of colonies after transformation (~ 20 colonies on average). Under these conditions, PCR screening of 50 positive colonies (Fig. 3C, Supplementary Fig. S3) demonstrate a success rate of 60%. However, repeated attempts at insertion the *atpI* and *ybbD* sites with ORF1p co-expression and 20 bp of 5’ and 40 bp of 3’ payload homology to target have so far proven unsuccessful. As with 3’ homology, further optimization of the amount of 5’ homology to the target included in the payload may improve the efficiency of insertion at low copy number targets. Inclusion of homology in the 5’ end of the payload, with the same sequence as the sgRNA, suggests the possibility of using a single RNA as both guide and payload. However, attempting to include the necessary secondary structure and using the 5’ end of the payload itself as the sgRNA for the Cas component proved unsuccessful, and we found it was necessary to co-transform two separate sgRNA and payload RNAs for successful targeted integration.

### Off-target effects and application to complex organisms

**Figure 4:**
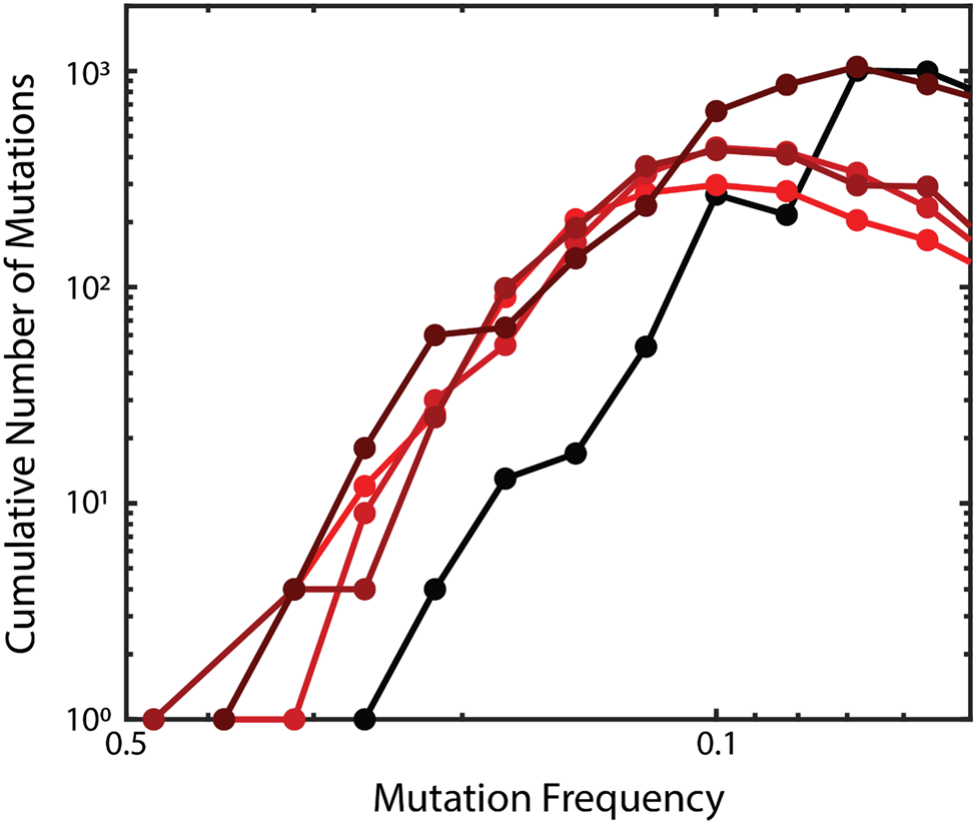
Cumulative number of mutations identified relative to the BL21-AI complete genome sequence (Accession NZ_CP047231.1, GI: 1797637028) for the BL21-AI negative control (black) and four replicates of BL21-AI subjected to the GENEWRITE expression.

Whole genome sequencing of cells subjected to the GENEWRITE expression exhibit larger numbers of high frequency mutations relative to a negative control, with mutations scattered throughout the genome (Fig. 4). Moreover, a large fraction of the plasmids purified and sequenced from GENEWRITE-exposed cells have curiously had the coding sequences of both GENEWRITE and NHEJ proteins excised from their host plasmids (Supplementary Fig. S4), suggesting that GENEWRITE may also be effective in excising coding regions of inappropriately-highly expressed genes.

## Discussion

We have shown that a fusion of a Cas endonuclease and LINE-1 ORF2p reverse transcriptase, which we call GENEWRITE, is capable of integration of large genetic payloads in the *E. coli* genome through appropriate design of the homology regions of guide and payload RNAs. We also find that assistance from NHEJ DNA repair enzymes and LINE-1 ORF1p protein may help increase the efficiency and specificity of the insertion (results summarized in Supplementary Table S3). We have not yet tested the limits on size of GENEWRITE payloads, but LINE-1 itself is ~ 5 kbp long and hence similarly sized payloads may be accessible.

The above-described results were obtained using a simplistic method where each component is delivered separately: the RNAs through electroporation, and the GENEWRITE protein through constitutive, low-level expression from a plasmid. We find GENEWRITE to be remarkably successful given this simplistic approach, despite ORF2p’s *cis*-preference for its encoding RNA^56^ and propensity to produce inserts with 5’ truncations^65–68^. However, using this method, we find significant off-target effects, including an increase in the rate of off-target mutations relative to a control, and the excision of highly expressed DNA segments from the genome. We speculate that these off-target effects and the lethality of the GENEWRITE protein to *E. coli* may be coupled: high affinity of ORF2p to arbitrary RNAs may force non-sgRNAs into the Cas component, serving as a guide for endonuclease activity and generating off-target DNA breaks. Hence, we suggest that rather than direct in vivo expression, deployment of the GENEWRITE system to more complex mammalian cells may be better accomplished through in vitro assembly and transfection of RNP particles as is frequently performed with traditional CRISPR-Cas editing. Furthermore, it remains to be seen if GENEWRITE insertions will produce truncated insertions when applied to more advanced systems.

## Methods

### Reagents

Primers used for PCR and RNA synthesis were synthesized by Integrated DNA Technologies (IDT, Coralville, IA). Kits used include QIAprep Spin Miniprep Kit (QIAGEN; Germantown, MD; Catalog Number 27106), QIAquick PCR Purification Kit (QIAGEN; Catalog Number 28106), DNeasy UltraClean Microbial Kit (QIAGEN; Catalog Number 12224-50), Megascript T7 Transcription Kit (ThermoFisher Scientific; Waltham, MA; Catalog Number AMB13345), TURBO DNase (ThermoFisher Scientific; Catalog Number AM2239), and NEBNext Ultra II Library Prep kit (New England Biosciences; Ipswich, MA; Catalog Number E7645S). PCR was performed with Phusion High-Fidelity PCR Master Mix with HF Buffer (NEB; M0531L).

### Biological resources and media

*E. coli* BL21-AI (ThermoFisher Scientific; Catalog Number C607003, GenBank accession number CP047231) was used for all experiments. Overnight seed cultures were grown in Super Optimal Broth with Catabolite Repression [SOC; SOB + 0.5% w/v glucose] medium with appropriate antibiotics. Electrocompetent cells were prepared by growth in Super Optimal Broth (SOB) with appropriate antibiotics.

### Plasmid design and construction

All GENEWRITE proteins and variants were designed in Vector NTI software (Thermo Fisher Scientific) and synthesized de novo and cloned into pUC57-*kan* by GENEWIZ Gene synthesis (GENEWIZ); the exception is ORF2pZRT, which was cloned into pUC57-*amp* by GENEWIZ. A list of all constructs used in this study is found in Supplementary Table S1.

*Bacillus subtilis* NHEJ enzymes [Ku (encoded by the gene *ykoV*) and LigD (encoded by the gene *ykoU*)] were expressed from the anhydrotetracycline-inducible P_LtetO1_ promoter^54^ on the plasmid pZA31^49^. Cells not expressing NHEJ were transformed with empty pZA31 as a control.

### sgRNA and payload RNA synthesis

DNAs encoding sgRNAs were prepared using primers including a T7 promoter driving a 20 bp guide sequence. The 3’ end of this primer was designed with a 14 bp overhang homologous to a 77 bp scaffold oligo containing sequence encoding the necessary sgRNA secondary structure and used to prime amplification of the sgRNA-encoding DNA. Sequences of all oligos used in the study are available in Supplementary Table S2.

Payload RNAs were prepared using primers including a T7 promoter driving sequence encoding a strong, constitutive PlacIQ1 promoter, a Shine-Dalgarno ribosomal binding site, and 20 bp of sequence homologous to the spectinomycin resistance gene *aadA*. Reverse primers were designed with 20 bp homology to the 3’ end of *aadA* and included indicated lengths of sequence homologous to the intended integration site. Payload RNA-encoding DNA was amplified from the plasmid pTKRED^69^ using these primers.

RNAs were generated using the above DNA templates using T7 MEGAscript (Thermo Fisher Scientific), with incubation at 37 °C for 16 h. Samples were then digested with TURBO DNase (Thermo Fisher Scientific) for 1 h at 37 °C and purified by phenol–chloroform extraction and isopropanol precipitation.

### Preparation of electrocompetent cells and transformation

Electrocompetent cells were prepared by preparation of a seed culture by overnight growth in SOC at 37 °C in a shaking water bath (New Brunswick C76). Seed cultures were diluted 10:1 in fresh SOB and grown at 37 °C in a shaking water bath until OD600 ~ 0.6, at which point 0.1% L-arabinose was added. Importantly, after L-arabinose was completely dissolved, cells were immediately harvested by centrifugation at 4 °C; extended induction of GENEWRITE with L-arabinose is lethal. This was followed by 3 × washing in ice-cold 10% v/v glycerol. 100 µl of cells thus prepared were mixed with an excess of payload RNA and sgRNA (5 μg and 10 μg, respectively); these quantities yielded success but have not been optimized. The mixture was electroporated (BIO-RAD Gene Pulser) using standard settings for *E. coli*. 1 ml of SOC + 1 mM IPTG and 100 ng/ml aTc were added, and cells were allowed to recover overnight. Transformants were spread on LB agar plates containing 100 µg/ml spectinomycin, 0.5% w/v glucose, 1 mM IPTG, and 100 ng/ml aTc and then incubated overnight in a 37 °C air incubator.

### Genome sequencing

Genomic DNA was obtained from cultures prepared in 2 ml Lysogeny Broth (LB) by purification using the QIAGEN DNeasy UltraClean Microbial Kit. Resulting samples were submitted to the UCR Genomic Core at the Institute for Integrative Genomic Biology for processing and sequencing. Samples were sheared using a Covaris S220 Ultrasonicator and libraries prepared using an NEBNext Ultra II Library Prep kit. After preparation, libraries were analyzed using qPCR and an Agilent 2100 Bioanalyzer. The resulting libraries were pooled and sequenced using an Illumina MiSeq sequencer with 150 bp paired-end reads. Sequencing data were analyzed with Geneious Prime.

## Supplementary Information

Supplementary Information.

## Data Availability

All data and materials are available upon reasonable request to thomas.kuhlman@ucr.edu. Sequence data are available at the following accession numbers: Cas9-GENEWRITE: MZ493917; Cpf1-GENEWRITE: MZ493918; Whole genome sequencing data: BioProject PRJNA743056.
