# Supplementary material for: Targeted insertion of large genetic payloads using cas directed LINE-1 reverse transcriptase
Source: Sci Rep. 2021 Dec 8;11:23625. doi: 10.1038/s41598-021-03130-0 (PMC8654924; doi:10.1038/s41598-021-03130-0)
Supplement: Supplementary file 1 — Supplementary Information. [file 41598_2021_3130_MOESM1_ESM.pdf]

Targeted Insertion of Large Genetic Payloads Using Cas Directed LINE-1 Reverse Transcriptase

Femila Manoj, Laura W. Tai, Katelyn Sun Mi Wang, and Thomas E. Kuhlman

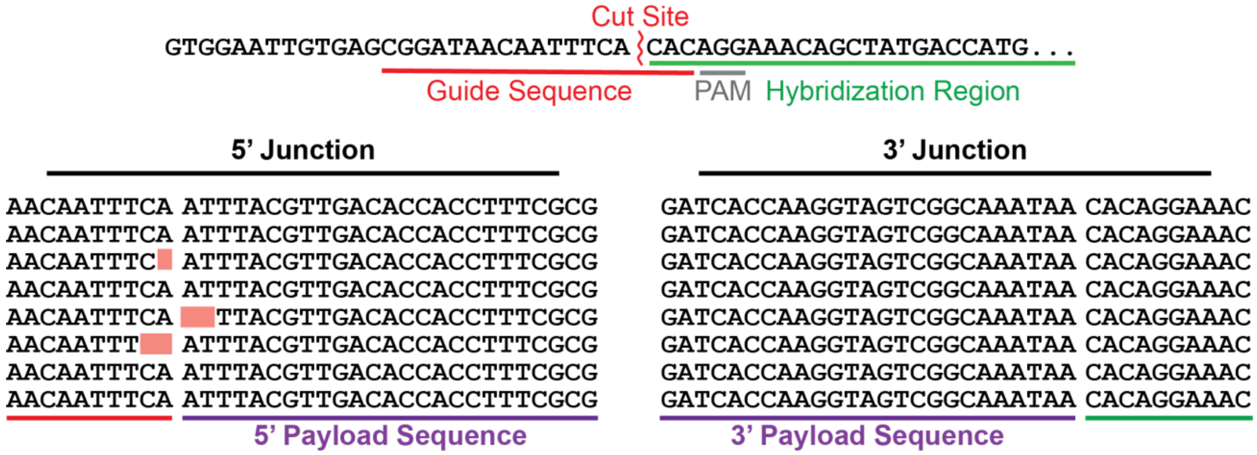

Supplementary Fig. S1.

Sequencing of eight positive colonies with insertions in pUC57-kan. Top: Sequence of target site and design features. Note that guide sequence+PAM is destroyed upon successful integration. Bottom: Sequencing of eight positive clones, with mismatches highlighted in red.

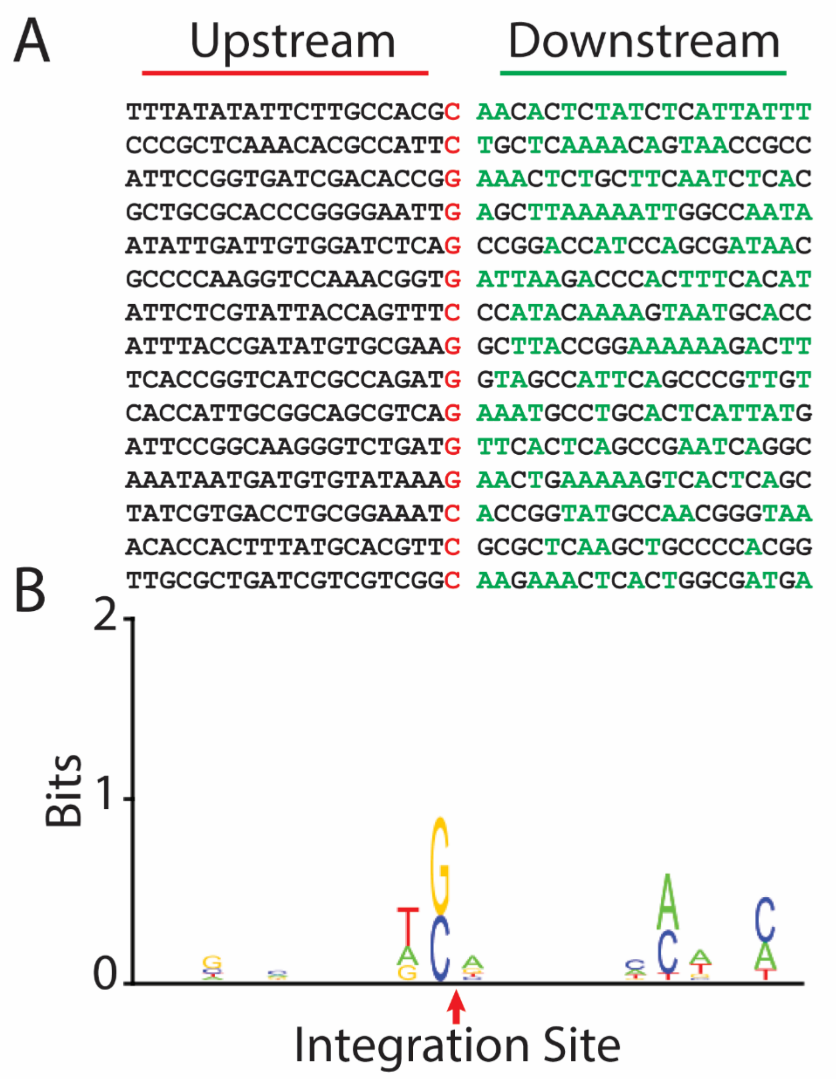

Supplementary Fig. S2

Insertion sites for LINE-1 retrotransposition in *E. coli*. 12 LINE-1 integration sites in *E. coli* identified by Illumina sequencing with 150 bp paired-end reads. (A) Sequences upstream and downstream of identified insertion locations. C/G immediately upstream of insertion is highlighted red, TA-rich regions downstream are highlighted green. (B) Logo plot of 20 bp surrounding integration site. Note G/C immediately upstream of insertion site is most prominent feature. In these experiment, LINE-1 was expressed in *E. coli* from a T7 promoter, and hence the first two basepairs at the 5’ end of the transcript are GC.

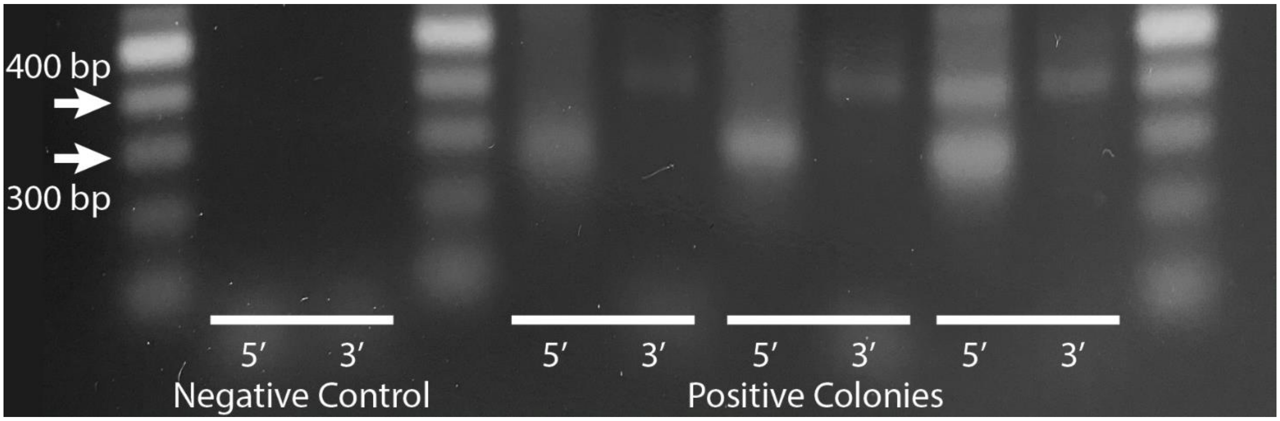

Supplementary Fig. S3.

PCR amplification across junctions created by GENEWRITE insertion at the *nth* chromosomal locus with 2% agarose gel electrophoresis. Amplicons expected from amplification across 5’ junction is 290 bp, 3’ junction is 400 bp.

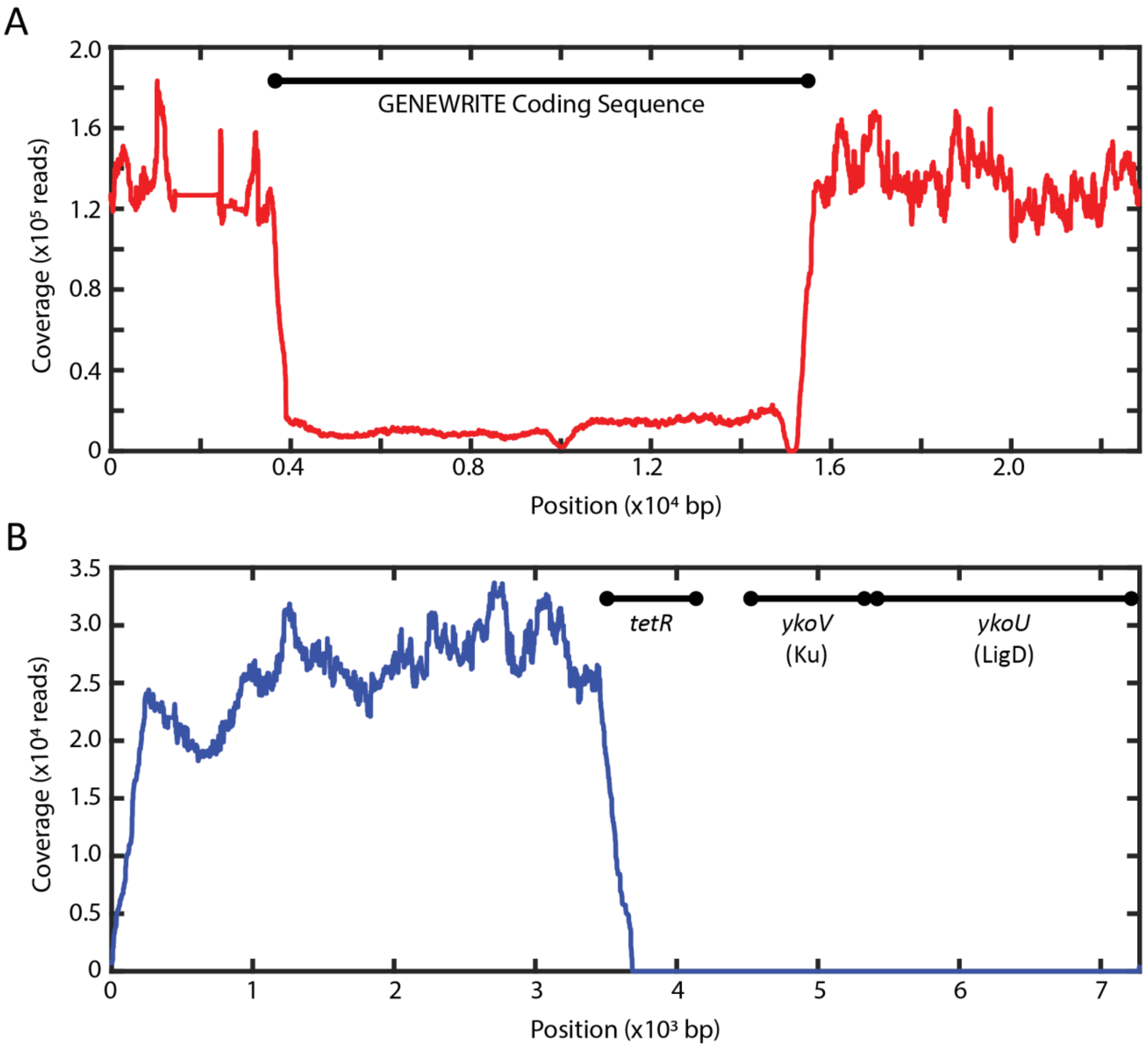

Supplementary Fig. S4.

Sequencing coverage of (A) pUC57-kan-GENEWRITE and (B) pZA31-NHEJ, illustrating the excision of coding sequences of strongly expressed genes from these plasmids. Regions corresponding to each coding region are indicated by labelled lines.

Plasmid	Antibiotic Resistance	Origin of Replication	Expressed Protein(s)	NLS	EN	Z	RT	NLS	6x His
pUC57-kan	Kanamycin (25 ug/ml)	pUC (~500 - 1000 copies/cell)	GENEWRITE (Cas9)	X	X (Cas9)	X	X	X	X
			GENEWRITE (Cas12a/Cpf1)	X	X (Cpf1)	X	X	X	X
			Cas9-ORF2pZRT		X (Cas9)	X	X		
			Cpf1-ORF2pZRT		X (Cpf1)	X	X		
			Cas9-ORF2pZRT + ORF1		X (Cas9)	X	X		
			Cas9Z		X (Cas9)	X			
pUC57-amp	Ampicillin (100 ug/ml)	pUC (~500 - 1000 copies/cell)	ORF2pZRT			X	X		
			ORF2pZRT -GAL4			X	X		
pZA31	Chloramphenicol (34 ug/ml)	p15A (~15 copies/cell)	NHEJ (ykoV + ykoU)						
			EMPTY						

Supplementary Table S1: GENEWRITE Constructs

Variants of GENEWRITE constructs used in this study.

Supplementary Table S2: Oligos used in this study.

**sgRNA oligos**	
scaffold	GTTTTAGAGCTAGAAATAGCAAGTTAAAATAAGGCTAGTCCGTTATCAACTTGAAAAAGTGGCACCGAGTCGGTGCT
scaffold R	AGCACCGACTCGGTGCCAC
T7 Cas9 pUC guide F	TAATACGACTCACTATAGGCGGATAACAATTTCACACGTTTTAGAGCTAGA
T7 Cpf1 pUC guide F	TAATACGACTCACTATAGCTGTGTGAAATTGTTATCCGGTTTTAGAGCTAGA
T7 pZA31 guide F	TAATACGACTCACTATAGGGCGAAAATGAGACGTTGATGTTTTAGAGCTAGA
T7 atpI guide F	TAATACGACTCACTATAGAATATCAGTCTGCTAAAAATGTTTTAGAGCTAGA
T7 nth guide F	TAATACGACTCACTATAGTGTCAGTGTTAATAAGGCGAGTTTTAGAGCTAGA
T7 ybbD guide F	TAATACGACTCACTATAGCTGACTGAGAAAAGACATGTGTTTTAGAGCTAGA
**Payload Oligos**	
T7-PlacIQ1-RBS-aadA F	TAATACGACTCACTATAGATTTACGTTGACACCACCTTTCGCGTATGGCATGATAGCGCCCGGAAGAGAGTCAATTCAGGAGGTAAATAATGCGCTCACGCAACTGGTCCAGAA
T7-pZA31-PlacIQ1-RBS-aadA F	TAATACGACTCACTATAGGGCGAAAATGAGACGTTGATATTTACGTTGACACCACCTTTCGCG
T7-atpI-PlacIQ1-RBS-aadA F	TAATACGACTCACTATAGAATATCAGTCTGCTAAAATTTACGTTGACACCACCTTTCGCG
T7-ybbD-PlacIQ1-RBS-aadA F	TAATACGACTCACTATAGCTGACTGAGAAAAGACATGTATTTACGTTGACACCACCTTTCGCG
T7-nth-PlacIQ1-RBS-aadA F	TAATACGACTCACTATAGTGTCAGTGTTAATAAGGCGAATTTACGTTGACACCACCTTTCGCG
pUC_lacZ0 R	TTATTTGCCGACTACCTTGGTGATC
pUC_lacZ10 R	GTTTCCTGTGTTATTTGCCGACTACCTTGGTGATC
pUC_lacZ20 R	GGTCATAGCTGTTTCCTGTGTTATTTGCCGACTACCTTGGTGATC
pUC_lacZ30 R	CCTCGAGCATGGTCATAGCTGTTTCCTGTGTTATTTGCCGACTACCTTGGTGATC

pUC_lacZ40 R	TTGGCTCGAGCCTCGAGCATGGTCATAGCTGTTTCCTGTGTTATTTGCCGACTACCTTGGTGATC
pUC_lacZ50 R	CGCGCCGAGCTTGGCTCGAGCCTCGAGCATGGTCATAGCTGTTTCCTGTGTTATTTGCCGACTACCTTGGTGATC
pUC_lacZ0pA R	TTTTTTTTTTTTTTTTTTTTTTTTTTTTTTTTATTTGCCGACTACCTTGGTGATC
pUC_lacZ10pA R	TTTTTTTTTTTTTTTTTTTTTTTTTTTTTTGTTTCCTGTGTTATTTGCCGACTACCTTGGTGATC
pUC_lacZ20pA R	TTTTTTTTTTTTTTTTTTTTTTTTTTTTTTGGTCATAGCTGTTTCCTGTGTTATTTGCCGACTACCTTGGTGATC
pUC_lacZ30pA R	TTTTTTTTTTTTTTTTTTTTTTTTTTTTTTCCTCGAGCATGGTCATAGCTGTTTCCTGTGTTATTTGCCGACTACCTTGGTGATC
pUC_lacZ40pA R	TTTTTTTTTTTTTTTTTTTTTTTTTTTTTTTTGGCTCGAGCCTCGAGCATGGTCATAGCTGTTTCCTGTGTTATTTGCCGACTACCTTGGTGATC
pUC_lacZ50pA R	TTTTTTTTTTTTTTTTTTTTTTTTTTTTTTCGCGCCGAGCTTGGCTCGAGCCTCGAGCATGGTCATAGCTGTTTCCTGTGTTATTTGCCGACTACCTTGGTGATC
aadA pZA31 hybridization R	AGTGATCTTATTTCATTATGGTGAAAGTTGGAACCTCTTACGTGCCGATCTTATTTGCCGACTACCTTGGTGATC
aadA atpI hybridization R	GACATTTTTAATAATGTTTTAACAGCCAATGATGGTTCTTAGCGCCGATTTTATTTGCCGACTACCTTGGTGATC
aadA nth hybridization R	TTCAAGCATCGCTGCAGGCGTATTCGCCACCGGGTAGAGTTTCGCCGTCGTTATTTGCCGACTACCTTGGTGATC
aadA ybbD hybridization R	CGAGTAGATATTCATCGTCTGAGCTATATGGCTTTACACAATAGCCGACATTATTTGCCGACTACCTTGGTGATC
aadA Cpf1 pUC hybridization R	CCCCAGGCTTTACACTTTATGCTTCCGGCTCGTATGTTGTGTGGAATTGTGAGCTTATTTGCCGACTACCTTGGTGATC
**Verification and Sequencing Oligos**	
aadA ver R	ACTGTACAAAAAAACAGTCATAAC
aadA ver F	CAGGCTTATCTTGGACAAGAAG
pUC ver F	AAACATCCCAATGGCGCGCCG
pUC ver R	GGCTTTACACTTTATGCTTC

pZA31 ver F	CGATAACTCAAAAAAATACG
pZA31 ver R	GACGTCGATATCTGGCGAA
atpI ver F	CTTCGTCAGGTGCAACATGAGC
atpI ver R	CAGTAACTGAACGAGCAGAAG
nth ver F	ACCACCGAGCTTAATTTCAGTTCGC
nth ver R	CCTGTTCGACGTTTTTCCCCGGCGC
ybbD ver F	ATTGGAGCTGGATTGCCTGATGCTTG
ybbD ver R	CTCACATTAAACACGTAACATTTTAATTAATG

Supplementary Table S3: Summary of Results

Insertion Target	Target Copy Number (per cell)	3' Homology	5' Homology	ORF1p Coexpression	Efficiency
pUC57-*kan*	500 - 1000	x			72%
pZA31	20 - 30	x			20%
E. coli chromosome (*nth* locus)	1 - 2	x			0%
		x	x		0%
		x		x	0%
		x	x	x	60%
